# Quality of life, delinquency and psychosocial functioning of adolescents in secure residential care: testing two assumptions of the Good Lives Model

**DOI:** 10.1186/s13034-017-0209-9

**Published:** 2018-01-08

**Authors:** C. S. Barendregt, A. M. Van der Laan, I. L. Bongers, Ch. Van Nieuwenhuizen

**Affiliations:** 1 Research and Documentation Centre (WODC) of the Dutch Ministry of Justice and Security, PO Box 20301, 2500 EH The Hague, The Netherlands; 2 GGzE Center for Child & Adolescent Psychiatry, PO Box 909 (DP 8001), 5600 AX Eindhoven, The Netherlands; 3 Scientific Center for Care & Welfare (Tranzo), Tilburg University, PO Box 90153, 5000 LE Tilburg, The Netherlands

## Abstract

**Background:**

In this study, two assumptions derived from the Good Lives Model were examined: whether subjective Quality of Life is related to delinquent behaviour and psychosocial problems, and whether adolescents with adequate coping skills are less likely to commit delinquent behaviour or show psychosocial problems.

**Method:**

To this end, data of 95 adolescents with severe psychiatric problems who participated in a four-wave longitudinal study were examined. Subjective Quality of Life was assessed with the ten domains of the Lancashire Quality of Life Profile and coping skills with the Utrecht Coping List for Adolescents.

**Results:**

Results showed that adolescents who reported a lower Quality of Life on the health domain had more psychosocial problems at follow-up. No relationship was found between Quality of Life and delinquent behaviour. In addition, active and passive coping were associated with delinquent behaviour and psychosocial functioning at follow-up.

**Conclusions:**

Based on the results of this longitudinal study, the strongest support was found for the second assumption derived from the Good Lives Model. Adolescents with adequate coping skills are less likely to commit delinquent behaviour and have fewer psychosocial problems at follow-up. The current study provides support for the use of strength-based elements in the treatment programmes for adolescents in secure residential care.

## Background

It is well established that criminogenic risks, such as age at first offense and number of prior convictions, predict later offending behaviour [1, 2]. As a consequence (juvenile) offender rehabilitation has primarily been focused on mapping and managing risks in the lives of delinquent adolescents. Herein, the Risk-Need-Responsivity (RNR) Model has for years been regarded as the standard approach in offender rehabilitation and therefore the most widely used rehabilitation theory [3]. The main underlying assumption of a risk management approach such as the RNR-Model, is that every individual that has offended in the past carries a risk for future reoffending [3]. By adhering to three main RNR principles (i.e., the risk principle, the need principle, and the responsivity principle) during treatment, this risk of reoffending can be decreased. The risk perspective in offender rehabilitation has been criticised for a number of reasons. First, it has been argued that the one-sided view of risk management does not allow for a more positive way of living and there is a lack of interest for positive indicators that might change behaviour [4]. Second, within the risk perspective in offender rehabilitation, a predominant ‘one size fits all’ mentality is apparent, with little attention for individual needs, skills and abilities [5]. In line with this, the risk perspective has also been criticised for its failure to motivate and engage offenders in their rehabilitation process [5]. In recent years, a shift has taken place from a risk-oriented view of offender rehabilitation towards a more strength-based rehabilitation view in which individuals’ needs, abilities and skills take a central role [3, 6]. Instead of looking at offenders as an accumulation of risks, they are seen as individuals who want to give meaning to their lives like any other person [6].

Alternative rehabilitation theories, such as the Good Lives Model, have been proposed and have been labelled ‘strength-based’ or ‘restorative’ approaches in working with individuals who have offended [3, 5]. This shift in offender rehabilitation can (at least partly) be attributed to several other findings. First, a large proportion of youngsters reoffended after they had received treatment in secure residential care [7–9]. This finding suggests that there is considerable scope for improvement in working with delinquent adolescents [3]. Second, there is a growing number of studies that identify factors other than risk factors that are associated with successful interventions and rehabilitation programmes, for example, subjective well-being and employment [e.g., [10–12]. Finally, especially for adolescents and young adult offenders, strength-based rehabilitation can be helpful guiding them in becoming healthy-functioning and productive adults [13].

The Good Lives Model operates according to a strength-based or restorative perspective in which the underlying processes of healthy functioning are the primary objects of treatment instead of those that underlie dysfunctional behaviour. Why and how adolescents desist from their criminal careers cannot be explained by risk factors alone. Other factors, such as meeting individual needs, improving Quality of Life (QoL), and developing coping skills might also be related to decreasing the risk of reoffending [6]. The Good Lives Model can be seen as a holistic approach that combines both the management of risk with the promotion of an offender’s well-being [4, 14]. According to the Good Lives Model, treatment should focus on the potential of an offender rather than emphasizing their incapacities and risk factors. From this holistic perspective, treatment is not only directed at decreasing the risk for reoffending but also to increasing an individuals’ psychosocial well-being. In addition, individuals should be engaged in productive activities in which they can learn and enhance skills, such as coping skills, that might help them in achieving their life goals. When individuals get the opportunity to create good and fulfilling lives for themselves, their individual risk of reoffending will decrease [4, 5]. Accordingly, a good and fulfilling life can be created by securing meaningful needs (i.e., primary human needs). The Good Lives Model proposes 11 groups of needs: (1) life, (2) knowledge, (3) excellence in work, (4) excellence in play, (5) excellence in agency, (6) inner peace, (7) relatedness, (8) community, (9) spirituality, (10) happiness, and (11) creativity [4, 6, 14]. It is assumed that each human being seeks these needs to some degree throughout their lives, although individual differences might exist. Fulfilling these needs in a socially acceptable manner will lead to an increase in an individuals’ subjective QoL and might also decrease the likelihood of reoffending.

Compared to the abundance of empirical studies that have been conducted with regard to risk factors in offender rehabilitation, relatively few studies have focused on the long term effects of securing needs, thereby increasing an individuals’ subjective QoL, and strengthening skills during treatment. In this paper, the focus will be on two concepts that both play a significant role in the Good Lives Model, namely subjective QoL and coping. Although the Good Lives Model acknowledges the importance of risk reduction, it also has a strong focus on the enhancement of an offender’s well-being or QoL. In daily practice, the enhancement of an individual’s QoL translates into identifying individuals’ priority needs in life and devising a good lives plan during treatment. This good lives plan consists of internal and external skills, abilities and resources that will contribute to the success of the plan, thereby increasing an individuals’ subjective QoL. Subjective QoL is a multidimensional concept and focuses on a person’s overall sense of well-being and satisfaction with life [15–17]. Among adults, a higher subjective QoL is associated with better emotional adjustment after discharge from a secure care facility [10]. Low subjective QoL, on the other hand, might increase the likelihood of delinquent behaviour [10, 18, 19]. Thus, according to the Good Lives Model, it can be assumed that the fulfilment of individual needs as described in a personalized good lives plan, increases a person’s subjective QoL, while also attending to risk factors, and thereby decreasing the chance of reoffending.

Coping can be seen as an internal resource or ability an individual can be equipped with in order to realize the goals set in his or her good lives plan. After identifying and prioritizing the primary human needs, a next step in the treatment process is to fulfil those needs in a socially acceptable manner. Once individuals are lacking proper skills or capabilities, they might use delinquent behaviour to secure the needs described in their good lives plan. Coping, in general, refers to the cognitive and emotional-behavioural strategies individuals use in response to stress [20], and is found to be related to the well-being of incarcerated adolescents [21]. From a Good Lives Model’s point of view, adequate coping skills can help individuals deal with problems and stress that individuals might experience in trying to fulfill their needs. In addition, adequate coping skills can help institutionalized offenders to adjust to the restricted environment of secure residential care. An active coping strategy is, for example, exercising while self-imposed social isolation is an example of a passive coping strategy [22]. Research has shown that poor coping strategies predict behavioural and emotional problems, such as problems with alcohol, depressive symptoms, and delinquent behaviour [23, 24]. More specifically, passive coping in adolescents is associated with adjustment problems [25] and depressive symptoms [24], and predicts poor well-being among adolescent detainees [26]. Thus, from a Good Lives Model perspective, the assumption is that using inadequate coping strategies might hinder the success of an individuals’ good lives plan and might increase the chance of reoffending.

The aim of this study is to test the following two assumptions derived from the Good Lives Model: (1) a higher subjective QoL in secure residential care is related to less reported delinquent behaviour and psychosocial problems at follow-up, and (2) having adequate coping skills in secure residential care, such as active coping, is related to less reported delinquent behaviour and psychosocial problems at follow-up. Both assumptions are connected since having adequate coping skills can also enable adolescents to fulfil their primary human needs and therefore increase their subjective QoL.

## Methods

### Setting

Participants were recruited from ten secure residential care facilities throughout the Netherlands that varied in terms of security level. Adolescents could be admitted to youth forensic psychiatric hospitals, child and adolescent psychiatric hospitals, orthopsychiatric institutions or youth detention centres. Throughout this paper, we use the term ‘secure residential care’ to refer to these institutions. Secure residential care refers to the most intensive or restrictive type of youth care in the Netherlands. Care, guidance and treatment are offered in a secure environment. Although adolescents from different treatment facilities were included, they shared comparable problems in multiple life domains such as experiencing problems with their living situation and having difficulties managing their finances, as well as a high prevalence of psychiatric disorders.

### Participants

The sample consisted of 95 Dutch male adolescents with severe psychiatric problems and problems in multiple life domains (e.g., raised in a single parent family). All adolescents were admitted to secure residential care. Respondents’ overall mean age at admission to secure residential care was 16.1 years (*SD* = 1.0). At the time of the first assessment their mean age was 16.7 years (*SD* = .9). Adolescents were eligible for participation if they were 16, 17 or 18 years of age, and if time of admission would be longer than 3 months. Of the 95 adolescents, 52 adolescents (54.7%) were sentenced under Dutch juvenile civil law and 43 adolescents (45.3%) were sentenced under Dutch juvenile criminal law. One of the measures under the Dutch juvenile civil law is the family supervision measure. This supervision measure is applied when the development of an adolescent is at risk and their parents or other caretakers are not able to help. These adolescents display severe behavioural problems and often lack motivation for voluntary treatment. The Dutch juvenile criminal law encompasses the treatment and rehabilitation of adolescents who have committed a serious criminal offense. Adolescents sentenced under the Dutch juvenile criminal law either have a regular detention sentence or a mandatory treatment order. Furthermore, 79 adolescents (83.2%) indicated that they used drugs at least once during their lives. The most common psychiatric disorder was a disruptive behaviour disorder (DBD: *n* = 58; 61.1%). Adolescents were also diagnosed with a range of other presenting issues including autism spectrum disorder (ASD: *n* = 29; 30.5%), attention deficit hyperactivity disorder (ADHD: *n* = 24; 25.3%), reactive attachment disorder (RAD: *n* = 14; 14.7%) and intellectual disability (ID: *n* = 17; 17.9%). In addition, it was known that 23 adolescents (24.2%) had debts during the Time 1 assessment and 57 adolescents (60.0%) indicated that their parents were divorced. More than half of the adolescents (*n* = 51; 53.7%) had failed a grade in school at least once.

## Measures

### Predictor variables

The Dutch Youth version of the Lancashire Quality of Life Profile (LQoLP) was used to measure subjective QoL [27–29]. This semi-structured interview was conducted at Time 1, which was during stay in a secure residential care facility. The LQoLP consists of objective and subjective indicators of QoL and measures the adolescent’s satisfaction with different QoL domains. For the subjective QoL estimates, the domains ‘social participation’ (6 items), ‘health’ (7 items), ‘family relations’ (6 items), ‘living situation’ (4 items), ‘safety’ (5 items), and ‘finances’ (4 items) were assessed using a 7-point Likert scale, ranging from ‘1 = could not be worse’ to ‘7 = could not be better’. The domains ‘positive esteem’ (5 items) and ‘negative esteem’ (5 items) were measured by means of a modified version of the Self-esteem Scale [30], while the domains ‘framework’ (10 items) and ‘fulfilment’ (13 items) were assessed using a 3-point Likert scale. The ‘framework’ subscale measured the degree to which an adolescent could envision having a meaningful perspective in his life, and the ‘fulfilment’ subscale measured whether the adolescents also had a set of life goals. Both scales were measured by the Life Regard Index [31]. The following transformation was applied in order to compare the mean scale scores of the domains with a 3-point response category to those with a 7-point response category: *M’* = (*M*: 3) × 7 [*M’* = transformed mean score; *M* = raw mean scale score]. Psychometric properties of the LQoLP have been demonstrated to be good [27, 32, 33].

To measure coping, the Utrecht Coping List for Adolescents (UCL-A) was used [34]. This questionnaire had to be filled in by the adolescents themselves during the Time 1 assessment in secure residential care. The UCL-A consists of seven scales: ‘active problem solving’ (7 items), ‘distraction’ (8 items), ‘avoidance’ (8 items), ‘social support seeking’ (6 items), ‘depressive reaction’ (7 items), ‘expressing emotions’ (3 items), and ‘comforting thoughts’ (5 items). All items were scored on a 4-point Likert scale, ranging from ‘1 = seldom or never’, ‘2 = sometimes’, ‘3 = often’, and ‘4 = very often’, with higher scores indicating more frequent use of a coping strategy. Active coping consists of the mean scores of the scales ‘confrontation’ and ‘seeking social support’, and passive coping consists of the mean scores of the scales ‘avoidance’ and ‘depressive reactions’ [35].

The Structured Assessment of Violence Risk in Youth (SAVRY) [36] was used to measure the risk and protective factors. The SAVRY is a risk assessment instrument designed to assist clinicians in evaluating risk for violence in adolescents. If a SAVRY was not conducted by a clinician, it was filled in by the researchers for the purpose of this study. The SAVRY was administered around the Time 1 assessment, when adolescents were admitted to a secure residential care facility. The SAVRY consists of 24 risk items and 6 protective items. The risk items are divided over three risk domains: ‘historical’ (10 items), ‘social/contextual’ (6 items), and ‘individual’ (8 items). The historical items are static in nature, while the social/contextual and individual items are dynamic. The risk items were scored ‘0 = low’, ‘1 = moderate’, or ‘2 = high’, and the protective items were scored ‘0 = absent’ or ‘2 = present’. A total risk score was calculated by summing the scores of the historical, social/contextual, and individual domains and a protective score was calculated by summing the protective items. A higher score on the risk and protective items indicated the presence of more risks and/or protective factors.

### Outcome variables

The Youth Delinquency Survey was used to measure self-reported delinquency at follow-up (Time 4) [37]. This survey is produced by the Research and Documentation Centre (WODC) of the Dutch Ministry of Justice and Security. Self-reported delinquency was measured by means of Computer Assisted Self Interviewing (CASI), whereby adolescents were asked if and how often they had committed a number of offenses over the previous 12 months. The delinquency score is a multiplication of the number of serious and non-serious delinquent behaviour and the frequency of the delinquent behaviour in the past year. Non-serious delinquent behaviour (e.g., ‘vehicle vandalism’ and ‘shoplifting of goods to the value of less than 10 euro’s’) was scored 1, whereas serious delinquent behaviour (e.g., ‘burglary’ and ‘use of violence in order to commit theft’) was scored 3. In addition, the frequency of the delinquent behaviour in the past year was scored as follows. Non-serious offenses committed 1–4 times were scored 1, and offenses committed 5 times or more were scored 2. Serious offenses committed 1 time were scored 1, offenses committed 2–4 times were scored 2, offenses committed 5–10 times were scored 3, and offenses committed 11 times or more were scored 4.

The Strengths and Difficulties Questionnaire (SDQ) was used to measure the psychosocial problems at follow-up (Time 4) [38–40]. For the administration of the SDQ, the CASI method was also used. The SDQ consists of 25 items that can be allocated to five subscales: ‘emotional symptoms’, ‘conduct problems’, ‘hyperactivity-inattention’, ‘peer problems’, and ‘pro-social behaviour. Each item has to be scored on a 3-point scale with ‘0 = not true’, ‘1 = somewhat true’, and ‘2 = certainly true’. A total difficulties score can be calculated by summing the scores of the subscales emotional symptoms, conduct problems, hyperactivity-inattention, and peer problems. In the current study, only the total difficulties score was used, with higher scores on this scale indicating more problems in psychosocial functioning.

Descriptive information on the predictor and outcome variables are shown in Table 1.

**Table 1:** Descriptive information on predictor and outcome variables (*n* = 95)

Variables	*M*	*SD*	Range	α
Risk and protective factors				
Total risk score	17.83	5.3	5–33	
Protective score	7.83	2.2	2–12	
Predictor variables (Time 1)				
Coping				
Active coping	14.73	3.4	7.5–24.5	.84
Passive coping	14.16	3.0	8.0–23.0	.76
Subjective QoL domains				
Living situation	3.45	1.2	1.0–6.0	
Social participation	5.24	.7	3.0–6.7	
Finances	4.02	1.5	1.0–7.0	
Health	5.36	.7	3.0–6.6	
Family relations	5.83	1.0	2.2–7.0	
Safety	5.76	.7	3.6–7.0	
Positive esteem	6.61	.6	4.2–7.0	
Negative esteem	6.32	1.0	3.3–7.0	
Fulfilment	5.71	1.0	3.1–7.0	
Framework	6.35	.7	3.7–7.0	
Outcome variables (Time 4)				
Delinquency	19.20	30.9	0–137	
Psychosocial problems	10.49	5.9	1.0–27.0	

*QoL* quality of life

### Procedure

The current study was part of a prospective longitudinal study with four waves of data (i.e., Time 1, Time 2, Time 3, and Time 4). Prior to the start of the study, the Medical Ethics Committee for Mental Health Institutions in the Netherlands (Ref. No: NL29932.097.09 CCMO) and the Ministry of Justice and Security gave their approval. Inclusion criteria were (1) male, (2) adolescents who remained institutionalized for a minimum period of 3 months after the Time 1 assessment and, (3) finished primary school in the Netherlands or had sufficient Dutch language skills. There were no specific exclusion criteria. However, adolescents had to be able to participate during the assessment. For example, being floridly psychotic at the time of the assessment would lead to exclusion from the study.

A total of 228 adolescents in secure residential care were approached to participate in the study. Of these, 40 adolescents refused to participate or their parents did not sign informed consent, and 16 adolescents were unable to participate because they transferred to other institutions or were discharged before the first assessment. The total response rate at Time 1 was 75.4% (*N* = 172). Of these 172 participants, 95 (55.2%) also conducted the follow-up assessment. To investigate the potential impact of attrition, we tested for differences between participants who completed the first assessment and the follow-up assessment (*n* = 95) and participants who dropped out after the first assessment (*n* = 77). Adolescents who completed the first assessment and the follow-up assessment were more often diagnosed with an autism spectrum disorder (ASD) and with a reactive attachment disorder (RAD) (respectively: χ^2^ (1) = 4.289, *p* < .05; χ^2^ (1) = 7.428, *p* < .01). There were no other significant differences found between the participants and the dropouts.

For all adolescents, clinicians as well as group workers estimated whether an adolescent could be asked to participate in the study. Once professionals had agreed, an adolescent was approached for participation and informed about the content of the study by the researchers. In addition, adolescents received an information leaflet that contained relevant information regarding the study, disclosed in understandable language. Adolescents were told no repercussions would follow upon refusing participation in the study. After verbal and written explanation of the study was given, a written informed consent was obtained from each adolescent who agreed to participate. For participants under the age of 18, parents were also asked for written informed consent.

In the current study only juveniles with both the first assessment (Time 1) and the follow-up assessment (Time 4) were analysed. The Time 1 assessment was at age 16, 17 or 18 and all adolescents were admitted to secure residential care during this assessment. Mean duration of stay in a secure residential care facility at the Time 1 assessment was 7.5 months (*SD* = 7.7). The follow-up assessment (Time 4) was planned 12 months after discharge from a secure residential care facility. Adolescents who were discharged were either living independently, moved back in with their parents or still received some sort of support or assistance with their living circumstances. Due to prolonged treatment some adolescents remained institutionalized during the course of the study. For those adolescents who remained institutionalized, the follow-up assessment was planned during their continued stay in secure residential care. Time in months between the Time 1 assessment and the follow-up assessment did vary (*M* = 19.6 months, *SD* = 4.8, range 10–32 months). This variation was dependent on the duration of juveniles’ stay in secure residential care. For those juveniles who remained institutionalized, the follow-up assessment (Time 4) was carefully planned in order for the time in months between the Time 1 assessment and the follow-up assessment to be equal for the *admitted* and *discharged* juveniles (respectively *M* = 18.2 months, *SD* = 4.6; *M* = 20.4 months, *SD* = 4.7).

### Data analysis

First, Pearson correlations of the predictors and outcomes measures were calculated. Predictor variables that showed non-significant associations with the outcome measures were removed from further analysis. Level of significance was set at *p* < .05. Second, stepwise linear multiple regression analyses were performed. A total risk score and a total protective score were continuously entered in the linear regression analyses. To predict delinquency and psychosocial problems at follow-up four models were estimated, and for each model the predictors were entered in one block. Model 1 included whether juveniles were admitted or discharged from secure residential care at the Time 4 follow-up assessment. This variable was included since differences were found between these groups. Admitted adolescents were significantly older at admission to secure residential care [*F*(93) = 2.180, *p* < .05], were more often admitted under the Dutch juvenile criminal law [χ^2^ (1) = 31.381, *p* < .001], had a higher total risk score [*F*(93) = .068, *p* < .01], and were more often diagnosed with conduct disorder [χ^2^ (1) = 5.450, *p* < .05], and intellectual disability (χ^2^ (1) = 8.718, *p* < .01). Model 2 added the total risk score and the protective score of the SAVRY. Model 3 added active and passive coping as predictors. In Model 4, the subjective QoL domains were added to the model. Multicollinearity between the independent variables was not a problem since the VIF values were below 5 and tolerance was above .2. The plots showed that the assumptions for linearity and homoscedasticity were not violated. SPSS version 19.0 was used to perform the analyses.

## Results

### Correlation analysis

First, in order to identify the variables for use in the predictive model, we looked at correlations between the predictor variables (i.e., active and passive coping and the QoL domains) and the outcome variables (i.e., self-reported delinquency and psychosocial problems). Table 2 shows these bivariate correlations between the dependent and independent variables. Only those predictors that were significantly (*p* < .05) correlated with the outcome measures delinquent behaviour and psychosocial problems at follow-up were used in further analyses. Only active coping (*r* = − .25, *p* < .01) at the Time 1 assessment was significantly correlated with delinquency at follow-up (Time 4). Therefore, both passive coping and all of the subjective QoL domains were excluded from any further analyses with regard to the outcome measure delinquency. With regard to the second outcome measure, psychosocial problems at follow-up, passive coping (*r* = .37, *p* < .01) and the subjective QoL domains social participation (*r* = − .22, *p* < .05), health (*r* = − .28, *p* < .01) and fulfilment (*r* = − .25, *p* < .05) showed a significant correlation. Therefore, active coping and all non-significant subjective QoL domains were excluded from any further analyses with regard to the outcome measure psychosocial problems.

**Table 2:** Correlations between risks, coping, subjective QoL domains and self-reported delinquency and psychosocial problems (*N* = 95)

Variables	1	2	3	4	5	6	7	8	9	10	11	12	13	14	15
Total risk score	–														
Protective score	.32**	–													
Active coping	− .02	− .11	–												
Passive coping	− .11	− .04	.21*	–											
Living situation	− .01	.10	− .05	− .20*	–										
Social participation	− .16	− .09	.04	− .16	.31**	–									
Health	− .02	− .02	.08	− .13	− .04	.26*	–								
Finances	.15	.04	− .03	− .14	.08	.25*	.17	–							
Family relations	.11	− .16	− .08	− .44**	.15	.13	.05	.19	–						
Safety	.10	− .09	− .16	− .24*	− .11	.05	.16	.26*	.25*	–					
Positive esteem	− .01	− .01	− .06	− .22*	.06	− .05	.21*	.15	.06	.25*	–				
Negative esteem	.18	.13	− .20	− .44**	.12	− .03	.17	.10	.28**	.22*	.45**	–			
Fulfilment	.07	− .10	.09	− .37**	.28**	.40**	.20*	.19	.45**	.31**	.32**	.45**	–		
Framework	− .05	− .15	.32**	− .13	.08	.10	.03	− .02	.04	.20	.31**	.21*	.47**	–	
Delinquency	.17	.08	− .25*	− .03	− .10	− .12	− .08	.05	− .03	.16	.02	.11	− .06	.01	–
Psychosocial problems	.13	.08	.09	.37**	− .17	− .22*	− .28**	− .03	− .18	− .05	− .16	− .15	− .25*	− .10	.40**

* *p* < .05, ** *p* < .01

### Delinquency

A second step in the analyses was to test how well the predictor variables were able to predict the outcome variable by means of a stepwise linear regression analysis. Thus, we studied how much variance in the outcome variable delinquency could be explained by active coping. Due to the variety in time of discharge at the Time 4 assessment, we included a dummy variable in every first model. In addition, to account for the disadvantaged backgrounds of the adolescents, a total risk score and a protective score were added to every second model. Finally, active coping was added in the third model. In the first model, being admitted or discharged from secure residential care at follow-up did not explain any variance in delinquency at follow-up [see Table 3: Model 1: *R*^2^ = .001, adjusted *R*^2^ = − .010, *F*(1,93) = .057, *p* = .811]. In the second model, adding risk and protective factors explained .5% of the variance in delinquency at follow-up [Model 2: *R*^2^ = .037, adjusted *R*^2^ = .005, *F*(3,91) = 1.173, *p* = .324]; this model however was not significant. In model 3, adding active coping as a predictor to the model explained 5.4% of the variance in delinquency at follow-up [Model 3: *R*^2^ = .094 adjusted *R*^2^ = .054, *F*(4,90) = 2.337, *p* = .061]. In this final model, active coping was a significant predictor of delinquency at follow-up (β = − .240, *p* < .05). The use of active coping was related to a decrease in self-reported delinquent behaviour at follow-up.

**Table 3:** Linear regression to predict delinquency (*N* = 95)

Variable	Model 1			Model 2			Model 3		
	B	SE	*β*	B	SE	*β*	B	SE	*β*
Discharged	− 1.57	6.54	− .03	− 5.78	6.89	− .09	− 4.99	6.73	− .08
Total risk score				1.14	.67	.20	1.14	.66	.19
Protective score				.31	1.53	.02	− .07	1.50	− .01
Active coping							− 2.18	.92	− .24*
*Adjusted R* ^*2*^		− .01			.01			.05	
Δ*R*^*2*^					.02			.04	

*B* unstandardized coefficients, *SE* standard error, *β* standardized coefficients

* *p* < .05

### Psychosocial problems

As a third and final step we tested how much variance in the outcome measure psychosocial problems can be explained by passive coping and three of the subjective QoL domains. Again, we accounted for whether adolescents were discharged or not in the first model, and for risk and protective factors in the second model. Then, passive coping was added in the third model and the QoL domains social participation, health and fulfilment in the fourth model. In the first model, being admitted or discharged from secure residential care at follow-up did not explain any variance in psychosocial problems at follow-up [see Table 4: Model 1: *R*^2^ = .010, adjusted *R*^2^ = − .001, *F*(1,93) = .893, *p* = .347]. In the second model, adding risk and protective factors also did not explain any variance in psychosocial problems at follow-up [Model 2: *R*^2^ = .022, adjusted *R*^2^ = − .011, *F*(3,91) = .673, *p* = .571]. Adding passive coping to the third model explained 13.7% of the variance in psychosocial problems at follow-up [Model 3: *R*^2^ = .173, adjusted *R*^2^ = .137, *F*(4,90) = 4.718, *p* < .05]. In model 4, adding the subjective QoL domains social participation, health, and fulfilment to the model, explained 16.9% of the variance in psychosocial problems at follow-up [Model 4: *R*^2^ = .231, adjusted *R*^2^ = .169, *F*(7,87) = 3.724, *p* < .05]. In this final model, passive coping was a significant predictor of psychosocial problems at follow-up (β = .329, *p* < .05). This indicates that adolescents who use more passive coping strategies in their problem solving, reported more psychosocial problems at follow-up. Additionally, the subjective QoL domain health was also a significant predictor of psychosocial problems at follow-up (β = − .198, *p* < .05). Adolescents who were more satisfied with their health during their stay in secure residential care reported less psychosocial problems at follow-up.

**Table 4:** Linear regression to predict psychosocial problems (*N* = 95)

Variable	Model 1			Model 2			Model 3			Model 4		
	B	SE	*β*	B	SE	*β*	B	SE	*β*	B	SE	*β*
Discharged	1.18	1.25	.10	.77	1.33	.06	.86	1.23	.07	.74	1.22	.06
Total risk score				.10	.13	.09	.15	.12	.13	.14	.12	.13
Protective score				.13	.30	.05	.14	.27	.05	.10	.27	.04
Passive coping							.79	.19	.39***	.66	.21	.33**
Social participation										− .46	.90	− .06
Health										− 1.61	.80	− .20*
Fulfilment										− .43	.69	− .07
*Adjusted R* ^*2*^		− .00			− .01			.14			.17	
Δ*R*^*2*^					.01			.15			.03	

*B* unstandardized coefficients, *SE* standard error, *β* standardized coefficients

* *p* < .05, ** *p* < .01, *** *p* < .001

## Discussion

The aim of the present study was to test two assumptions derived from the Good Lives Model. First, it is assumed that a higher subjective QoL in secure residential care facility is related to less self-reported delinquency and psychosocial problems after discharge from the secure residential care facility. The current findings show that none of the subjective QoL domains were associated with delinquency. With regard to psychosocial functioning, the subjective QoL domain health was a significant predictor. Adolescents who reported a lower QoL on the health domain during their stay in a secure residential care facility had more psychosocial problems at follow-up. Second, it is assumed that having adequate coping skills during stay in a secure residential care facility, such as active coping, is related to less self-reported delinquency and psychosocial problems after having left the facility. The results of the current study support this assumption. Adolescents who used active coping strategies when facing a stressful or problematic situation while institutionalized reported less delinquent behaviour once they had left the facility.

The Good Lives Model places strong emphasis on the process of engaging individuals in their treatment by focusing on life goals and needs that are important to them. As a result, adolescents create a ‘good life’ for themselves, which is characterized by a sense of purpose, autonomy and a high QoL [3]. It is hypothesized that, due to increased feelings of agency and a higher QoL, adolescents are motivated to live a different kind of life and this will also help prevent them from re-offending [5]. However, the findings of the present study do not support this assumption, indicating that increasing the subjective QoL of adolescents who were institutionalized did not directly relate to a decrease in delinquency after they were discharged. A previous study among a sample of adult forensic psychiatric outpatients did find support for this assumption [10]. Adult forensic psychiatric outpatients who were more satisfied with their health reported less violent and general offenses. This difference in results might be due to the difference in the studied population and the context in which they resided during the time of the study. Whereas the current study examined adolescents that were admitted to a secure residential care facility and were treated for their emotional and behavioural problems, Bouman and colleagues studied adult forensic psychiatric outpatients, who did not receive treatment in a secured setting. Thus, it may be that the secure nature of the facility influenced the results of the current study. A second difference between both studies that might explain the difference in findings is that the current study included adolescents while Bouman and colleagues included adults. Adults and adolescents might differ in the weightings that they give to their primary human needs (i.e., their QoL domains). Specific needs that adults generally find very important might not be perceived as that important by adolescents and as a result also not strongly relate to delinquent behaviour or psychosocial well-being.

With regard to the second outcome variable psychosocial functioning we found a relationship with the subjective QoL domain health. This finding is comparable to other researchers that have studied these concepts in the general population [41]. Adolescents who reported to be more satisfied with their health during their stay in a secure residential care facility (e.g., being satisfied with their medicine use and their mental health), reported lower levels of psychosocial problems after they were discharged from that secure residential care facility. This finding remained even after controlling for the presence of risk factors and the use of active and passive coping strategies. Thus, once adolescents are more satisfied with their health during institutionalization, the likelihood that they will experience psychosocial problems after they leave the facility will decrease, regardless of the presence of risks or type of coping strategies used during their admittance.

Consistent with our expectations, adolescents who used adequate coping strategies during their admission in a secure residential care facility reported less delinquent behaviour and fewer psychosocial problems after they were discharged from that facility. These relationships were found regardless of whether adolescents had a disadvantaged background as indicated by the presence of multiple risk factors. According to the Good Lives Model, adolescents that are lacking adequate skills in order to secure needs that are meaningful to them will attempt to achieve these needs by (re-)offending [3]. The results of the present study support this assumption and are in line with the results of other studies [21, 23, 42]. Adolescents using active coping strategies (e.g., actively trying to sort out a problematic or stressful situation or seek social support with friends or family) during their stay in secure residential care reported less delinquent behaviour after they left the secured facility. Teaching adolescents the use of active coping skills during their institutionalization might decrease the chance that they will show delinquent behaviour again after their discharge. In addition, adolescents who used passive coping strategies, such as avoiding the problem or showing a depressive response when facing a problem or stressful situation, reported higher levels of psychosocial problems after leaving the facility. Previous studies also showed that the use of passive coping was associated with negative outcomes among adolescent prisoners, such as a reduced well-being [26] and increased psychological stress [43]. Our findings support the assumption derived from the Good Lives Model that a lack of adequate coping strategies is predictive of delinquent behaviour and psychosocial problems at follow-up, even after controlling for the presence of risk and protective factors.

The current study has a number of limitations that should be considered when interpreting the results. First, only self-report measures were used to assess delinquent behaviour and psychosocial functioning at follow-up. Although we considered both the severity of the offenses, as well as the number of offenses that were committed, it remains possible that the findings reported here under represent official registration data. Second, the current study is part of a longitudinal study with four waves of data. Adolescents were approached every 6 months to assess their subjective QoL during their stay in a secure residential care facility and also 12 months after discharge. The current study only used data from participants who completed the first assessment and the follow-up assessment. This way, only data was used of 95 of the 172 included adolescents. Attrition analysis revealed that these adolescents were more often diagnosed with an autism spectrum disorder (ASD) and with a reactive attachment disorder (RAD), which might cause results to be less generalizable.

## Conclusions

Subjective QoL and coping are important components of the Good Lives Model framework and are assumed to play a role in the onset and maintenance of delinquent behaviour and psychosocial problems [4, 6]. Strength-based approaches are increasingly used in the treatment of adolescents in secure residential care and might be an important complement to the prevailing risk perspective. By solely focusing on criminogenic risks as main treatment targets, other factors, such as subjective QoL and coping are neglected. The current study showed that adolescents who reported a lower QoL on the health domain had more psychosocial problems at follow-up. No relationship was found however, between QoL and delinquency. Based on the results of the current study, the strongest support was found for the second assumption derived from the Good Lives Model: adolescents with adequate coping skills report less delinquent behaviour and fewer psychosocial problems. Adolescents lacking adequate coping skills were more likely to experience adjustment problems upon returning to society. Adolescents who used active coping during their stay in secure residential care reported lower levels of delinquent behaviour at follow-up, while adolescents who used passive coping during their stay in secure residential care reported higher levels of psychosocial problems at follow-up. To conclude, we could not confirm the first assumption derived from the Good Lives Model in our sample of adolescents with severe psychiatric problems. However, results of this study provide support for the second assumption and therefore underline the importance of developing and strengthening adequate coping skills in the treatment of adolescents with severe psychiatric problems.

## References

[1] Farrington DP (2003). Developmental and life-course criminology: key theoretical and empirical issues—the 2002 Sutherland award address. Criminology.

[2] Stouthamer-Loeber M, Loeber R, Wei E, Farrington DP, Wikstrom POH (2002). Risk and promotive effects in the explanation of persistent serious delinquency in boys. J Consult Clin Psychol.

[3] Fortune C-A, Ward T, Willis GM (2012). The rehabilitation of offenders: reducing risk and promoting better lives. Psychiatry Psychol Law.

[4] Ward T, Gannon TA (2006). Rehabilitation, etiology, and self-regulation: the comprehensive Good Lives Model of treatment for sexual offenders. Aggress Violent Behav.

[5] Ward T, Marshall WL (2004). Good lives, aetiology and the rehabilitation of sex offenders: a bridging theory. J Sex Aggress.

[6] Purvis M, Ward T, Willis G (2011). The Good Lives Model in practice: offence pathways and case management. European Journal of Probation.

[7] Letourneau EJ, Armstrong KS (2008). Recidivism rates for registered and nonregistered juvenile sexual offenders. Sex Abuse.

[8] Mulder E, Vermunt J, Brand E, Bullens R, Van Marle H (2012). Recidivism in subgroups of serious juvenile offenders: different profiles, different risks?. Crim Behav Mental Health.

[9] Van Marle HJC, Hempel IS, Buck NML (2010). Young serious and vulnerable offenders in the Netherlands: a cohort follow-up study after completion of a PIJ (detention) order. Crim Behav Mental Health.

[10] Bouman YHA, Schene AH, De Ruiter C (2009). Subjective well-being and recidivism in forensic psychiatric outpatients. Int J Forensic Mental Health.

[11] Bahr SJ, Harris L, Fisher JK, Harker Armstrong A (2010). Successful reentry: what differentiates successful and unsuccessful parolees?. Int J Offender Ther Comp Criminol.

[12] Tripodi SJ, Kim JS, Bender K (2010). Is employment associated with reduced recidivism? The complex relationship between employment and crime. Int J Offender Ther Comp Criminol.

[13] Steinberg L, Chung HL, Little M (2004). Re-entry of young offenders from the justice system: a developmental perspective. Youth Violence Juv J.

[14] Ward T (2002). The management of risk and the design of good lives. Aust Psychol.

[15] Lehman AF (1996). Measures of quality of life among persons with severe and persistent mental disorders. Soc Psychiatry Psych Epidemiol.

[16] Lehman AF (1983). The well-being of chronic mental patients. Arch General Psychiatry.

[17] Reininghaus U, McCabe R, Burns T, Croudace T, Priebe S (2012). The validity of subjective quality of life measures in psychotic patients with severe psychopathology and cognitive deficits: an item response model analysis. Qual Life Res.

[18] Draine J, Solomon P (1992). Comparison of seriously mentally ill case management clients with and without arrest histories. J Psychiatry Law.

[19] Draine J, Solomon P (1994). Jail recidivism and the intensity of case management services among homeless persons with mental illness leaving jail. J Psychiatry Law.

[20] Compas BE, Connor-Smith JK, Saltzman H, Thomsen AH, Wadsworth ME (2001). Coping with stress during childhood and adolescence: problems, progress, and potential in theory and research. Psychol Bull.

[21] Gullone E, Jones T, Cummins R (2000). Coping styles and prison experience as predictors of psychological well-being in male prisoners. Psychiatry Psychol Law.

[22] Ashkar PJ, Kenny DT (2008). Views from the inside—young offenders’ subjective experiences of incarceration. Int J Offender Ther Comp Criminol.

[23] Mulder E, Brand E, Bullens R, Van Marle H (2011). Risk factors for overall recidivism and severity of recidivism in serious juvenile offenders. Int J Offender Ther Comp Criminol.

[24] Windle M, Windle RC (1996). Coping strategies, drinking motives, and stressful life events among middle adolescents: associations with emotional and behavioral problems and with academic functioning. J Abnorm Psychol.

[25] Ebata AT, Moos RH (1991). Coping and adjustment in distressed and healthy adolescents. J Appl Dev Psychol.

[26] Brown SL, Ireland CA (2006). Coping style and distress in newly incarcerated male adolescents. J Adolesc Health.

[27] Van Nieuwenhuizen C, Schene AH, Koeter MWJ, Huxley PJ (2001). The Lancashire quality of life profile: modification and psychometric evaluation. Soc Psychiatry Psych Epidemiol.

[28] Van Nieuwenhuizen C, Schene AH, Koeter MWJ (2002). Quality of life in forensic psychiatry: an unreclaimed territory?. Int Rev Psychiatry.

[29] Harder AT, Knorth EJ, Kalverboer ME (2011). Transition secured? A follow-up study of adolescents who have left secure residential care. Child Youth Serv Rev.

[30] Rosenberg M (1965). Society and the adolescent self-image.

[31] Debats DL, Van der Lubbe PM, Wezeman FRA (1993). On the psychometric properties of the life regard index (lri)—a measure of meaningful life—an evaluation in 3 independent samples based on the Dutch version. Pers Indiv Differ.

[32] Van Nieuwenhuizen C, Schene A, Boevink W, Wolf J (1998). The Lancashire quality of life profile: first experiences in the Netherlands. Commun Mental Health J.

[33] Oliver JPJ, Huxley PJ, Priebe S, Kaiser W (1997). Measuring the quality of life of severely mentally ill people using the Lancashire quality of life profile. Soc Psychiatry Psych Epidemiol.

[34] Bijstra JO, Bosma HA, Jackson S (1994). The relationship between social skills and psychosocial functioning in early adolescence. Pers Indiv Differ.

[35] Meijer SA, Sinnema G, Bijstra JO, Mellenbergh GJ, Wolters WHG (2002). Coping styles and locus of control as predictors for psychological adjustment of adolescents with a chronic illness. Soc Sci Med.

[36] Borum R, Bartel P, Forth A (2002). Manual for the structured assessment of violence risk in youth (SAVRY), consultation edition, version 1.

[37] Van der Laan AM, Blom M, Kleemans ER (2009). Exploring long-term and short-term risk factors for serious delinquency. Eur J Criminol.

[38] Goodman R (1997). The strengths and difficulties questionnaire: a research note. J Child Psychol Psychiatry.

[39] Goodman R (1999). The extended version of the strengths and difficulties questionnaire as a guide to child psychiatric caseness and consequent burden. J Child Psychol Psychiatry.

[40] Goodman R (2001). Psychometric properties of the strengths and difficulties questionnaire. J Am Acad Child Psychiatry.

[41] Bartels M, Cacioppo JT, van Beijsterveldt TCEM, Boomsma DI (2013). Exploring the association between well-being and psychopathology in adolescents. Behav Genet.

[42] Shulman EP, Cauffman E (2011). Coping while incarcerated: a study of male juvenile offenders. J Res Adolesc.

[43] Ireland JL, Boustead R, Ireland CA (2005). Coping style and psychological health among adolescent prisoners: a study of young and juvenile offenders. J Adolesc.

